# How Does Household Food Insecurity Impact Complementary Feeding, in High Income Countries, in a Cost‐of‐Living Crisis? A Systematic Scoping Review

**DOI:** 10.1111/mcn.70082

**Published:** 2025-08-20

**Authors:** Grace Hollinrake, Lowri Stevenson, Laura L. Wilkinson, Sophia Komninou, Amy Brown

**Affiliations:** ^1^ Faculty of Medicine, Health and Life Sciences Swansea University Singleton Park Swansea UK; ^2^ Lactation, Infant Feeding and Translational Research (LIFT) Research Centre Swansea University Swansea UK

**Keywords:** complementary feeding, household food insecurity, infant feeding, parents

## Abstract

Complementary feeding, when infants are introduced to solid foods, is an important stage of learning new tastes, textures and eating behaviours. Austerity, post‐BREXIT (in the UK) and the COVID‐19 pandemic have created a cost‐of‐living crisis, exacerbating prevalence of food insecurity in high‐income countries. Understanding how this may impact upon parents' experience of complementary feeding is important. This systematic scoping review therefore examined how food insecurity impacts diet and feeding practices during the complementary feeding period for infants aged 6–18 months. Four electronic databases were searched, identifying 5822 articles. 3293 titles and abstracts, from which 30 full texts were screened by two independent reviewers. The final review included five articles (two qualitative and three quantitative). Three articles were conducted in Australia, one in America, one in New Zealand with 1044 parent/child dyads in total. Strategies such as encouraging children to finish their food, avoiding foods that might not be accepted and reducing food variety were common. These strategies may ensure children are fed but may reduce elements of complementary feeding that we know are important such as exposing infants to wide varieties of tastes, textures and nutrients and adopting a responsive feeding style. The sparsity of evidence in this review, particularly for research based in the UK, highlights the need for further research in high‐income countries to explore the impact of household food insecurity on complementary feeding. This will help to identify priorities for those working in policy and practice to support families with complementary feeding during the cost‐of‐living crisis and beyond.

## Summary

- The impact of household food insecurity on complementary feeding was reviewed.
- Food insecure households were more likely to use strategies such as purchasing foods they knew would be accepted and feeding perceived high satiety foods.
- Food insecure households were more likely to use coercive feeding practices.
- Only one study focused on household food insecurity and the complementary feeding period.

## Graphical abstract

The impact of household food insecurity on complementary feeding, in high‐income countries, in the cost‐of‐living crisis was reviewed. Five studies were reviewed, only one study, from New Zealand, focused on household food insecurity and the complementary feeding period, specifically baby food pouches and feeding approach. More research is needed.

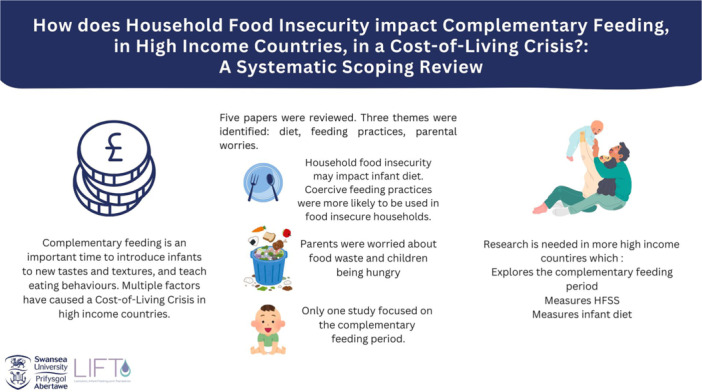

## 1 Background

The complementary feeding period, where infants transition from milk only to introducing solid foods and eating a family diet, is an important stage of learning new tastes, textures and eating behaviours (WHO 2023). It is recommended that infants are introduced gradually to solids from around 6 months old, to meet nutritional needs that milk alone can no longer provide (Emmett and Jones 2014; WHO 2023). Food choices should ideally be carefully chosen to ‘complement’ continued milk feeding, exposing the infant to a wide variety of foods and nutrients (WHO 2023).

Complementary feeding is an important time of learning tastes and textures. Introducing different foods, which are not only different from milk but different to each other, is an immediate shift from what the infant is used to, regarding texture and taste; how foods feel in the mouth and hands will be a new sensory experience (Mennella et al. 2016; Simione et al. 2018). These taste and texture learnings require several exposures to novel foods before being accepted, including tasting the food, not just seeing it (Ventura and Worobey 2013). Learning about new tastes and textures during infancy can track into later life and may influence food choices in childhood, reducing chance of fussy eating (Caton et al. 2014; Mennella et al. 2016; Ventura and Worobey 2013).

Eating behaviours are also learnt at this age with parents playing an important role in helping shape later food choices and eating styles. A responsive feeding style where infant cues of hunger and satiety are followed, can help infants learn when they are hungry and full, helping to support healthy weight trajectories (Brown and Lee 2015). Caregivers also help teach food choices through role modelling, eating meals together, eating the same foods and the social interactions that can occur at mealtimes (Elliott et al. 2023). These may track into later life and inform the expectations of mealtimes, and eating habits (Jansen et al. 2018; Yelverton et al. 2021).

Hence, complementary feeding is an important time for infants to learn new skills and learn about food. However, parents often feel anxious during this period, with concerns around what or how to feed their baby (Tully et al. 2019). There are many sociocultural, economic and psychological factors that can affect parents' concerns alongside the nutrients that infants receive and feeding styles (Arden 2010; Brown et al. 2017; Brown and Lee 2013; Wang et al. 2019). One important factor is income. Studies with older children are well established that poverty affects food choices (Berge et al. 2020; Burke et al. 2017; Evans et al. 2018; Hevesi et al. 2024). Research has highlighted that families who are experiencing poverty may worry about affordability of food, unmeasured monetary costs of food such as food waste, and housing and utility costs (Daniel 2020; Hayter et al. 2015; Pybus et al. 2021). Due to this food insecure parents may be less likely to frequently purchase fresh fruit and vegetables or provide an alternative meal for fussy eaters (Harris et al. 2019; Hutchinson and Tarasuk 2022). These studies were mainly conducted before the cost‐of‐living crisis. The cost‐of‐living crisis has created living conditions we have not previously seen, with more households becoming food insecure. Therefore, complementary feeding is likely being impacted in a way we have not seen previously, in terms of diet and feeding practices. However, little attention has been given to researching the effect of household food insecurity on complementary feeding during this cost‐of‐living crisis.

Many high‐income countries are currently experiencing a cost‐of‐living crisis. This is estimated to have begun in 2018 due to inflation which household incomes have not increased to meet (Hourston 2022). Recent drivers of inflation include climate disasters, long‐term austerity measures (defined as economic polices implemented to decrease government budget deficit but negatively impact the population), conflict (such as the war in Ukraine), changes in trade agreements (such as BREXIT), and the coronavirus‐19 (COVID‐19) pandemic. These all impact the complex global food system, such as impacting crop yield, supply chains and food imports. High inflation has directly and indirectly impacted food affordability, directly through increased food prices and indirectly through decreased employment rates and increased energy prices causing restricted household budgets (Devereux et al. 2020; Stone et al. 2024); making more households food insecure. Although there is not one agreed definition of household food insecurity, it is a multidimensional issue, where if households are food insecure this may impact access to food, the quality and/or quantity of food, and the emotional wellbeing and mental load of the people in the household (Dowler et al. 2001).

Rates of household food insecurity in high‐income countries are increasing (Foodbank 2024; The Trussell Trust 2023; Uppal 2023; US Department of Agriculture USDA 2025). Statistics from the UK show in 2023 there have been the highest levels of households accessing food banks, especially households with children (The Trussell Trust 2023). The Joseph Rowntree Foundation (2024) found poverty rates in families with children are highest in households with children 0–4 years old, in the UK. The Trussell Trust also reported children aged 0–4 years are at highest risk of living in hunger and hardship (Weekes et al. 2024). Additionally, there is not only increased rates of poverty but deepening of poverty in the UK, with more households living in destitution (JRF 2024). Furthermore, the latest calculations from the Trussell Trust show percentage of people experiencing hunger and hardship is likely to increase until 2026/27, meaning the situation is likely to get worse with more households experiencing food insecurity (Weekes et al. 2024). Moreover, households previously thought of as being financially secure as they are not low income, have self‐reported becoming food insecure, since the COVID‐19 pandemic (Thomas et al. 2022).

Research has shown food insecurity negatively impacts older children's food intake and feeding practices, including poor diet with lower consumption of fruit and vegetables, and children feeling hungry (Bell et al. 2023; Harvey 2016). There have been several systematic and scoping reviews on the topic of household food insecurity's impact on children, showing the importance of research in this area. For example, Baxter et al. (2022) conducted a scoping review of the literature up to 2021 and found most research is from America and Australia exploring household food insecurity's impact on feeding practices in 0–5 year olds. Moreover, Bell et al. (2023) explored the current literature in Europe up to 2021, on the impact of household food insecurity on children of all ages. However, they found no studies investigating household food insecurities impact on children under 18 months old (Bell et al. 2023). Furthermore, they focus on wider periods of economic crisis pre‐2021, such as the global financial crisis in 2008. The current cost of living crisis is more recent, affected by specific factors such as COVID‐19, BREXIT, political unrest and war. It appears more widespread, affecting all families through rapid inflation (JRF 2024). Food prices have been particularly affected in recent years (The Food Foundation and City University 2023).

Rates of household food insecurity have increased, and families with children under 5 years old are most impacted, this includes the complementary feeding period. However, it is largely unknown how household food insecurity impacts complementary feeding. Therefore, this systematic scoping review adds to the literature by providing a synthesis of the body of literature that examines the impact of household food insecurity on infants and young children's (6–18‐month‐olds), diet and feeding practices, in high‐income countries, in the current cost‐of‐living crisis (2018–2024). The aims of this systematic scoping review are:

1. To establish the existing body of knowledge around the impact of household food insecurity on complementary feeding, specifically diet and feeding practices.
2. To identify gaps in the literature to inform future research.

## 2 Methods

The review protocol was preregistered via the Open Science Framework before the literature search (https://osf.io/ts3d8).

### 2.1 Identifying the Research Question

The research question was developed using the PEO framework which stands for population, exposure and outcome(s) (Moola et al. 2015). The PICO framework was considered, which stands for Population, Intervention, Comparison and Outcome(s) (Richardson et al. 1995). Although, it was decided inappropriate as intervention is not of interest in this study, and the framework is commonly applied to form research questions for clinical reviews to inform evidence‐based practice (Richardson et al. 1995), which is not the purpose of this review. Hence, the PEO framework was chosen as it does not consider intervention and helps structure questions to identify the relationship, if any, between two variables (exposure and outcome), in a specific population (Moola et al. 2015).

For this systematic scoping review, it was considered how household food insecurity (exposure) impacts infant and young children's (population) diet and feeding practices (outcomes), in the cost‐of‐living crisis, in high‐income countries. The cost‐of‐living crisis is the rise in households unable or struggling to afford necessities to live such as rent, utilities, and food, due to increased costs, and loss of income and or employment, estimated to have begun in 2018.

### 2.2 Identifying the Relevant Studies

Inclusion and exclusion criteria (see Table 1) were created with the aid of the PEO framework, with additional factors considered. The age range of 6–18 months was chosen as complementary feeding is recommended to begin at around 6 months old (World Health Organisation 2023). It is more difficult to specify when the complementary feeding period ends, but it is recommended that young children are following an adult diet at around 2 years old (World Health Organisation 2023). Therefore, 18 months old was chosen as the oldest age as children are still likely to be in the complementary feeding period, hence 6–18 months covers the different stages of weaning, with the gradual increase in solid foods. Moreover, reviews focusing on children start their age range at 18 months old. Interventions and programmes which help food insecure households are of importance. However, for the purpose of this study these were excluded as we wanted to know the effect of household food insecurity, without such intervention.

**Table 1:** Inclusion and exclusion criteria of systematic scoping review.

	Inclusion	Exclusion
Person/population	Infants and young children aged 6–18 months	Studies restricted to a specific type of population not directly linked to infants and young children or with clinical needs which require a specific diet
		Perspectives of those outside the household such as healthcare professionals
Exposure	Food insecure household	Food secure populations
		Studies which evaluate food‐insecurity interventions or services
Outcomes	Diet	Experiences and accounts not specifically related to food
	Feeding practices, e.g., responsive feeding	
Study type	Qualitative studies	Reviews
	Quantitative studies	Expert opinion articles
	Mixed methods studies	Editorials
	Primary data sources from grey literature	Policy documents
		Conference abstracts
		Qualitative sources that are discourse analysis, analysed text
		Grey literature that does not include primary data
Study period	Since 2018	Studies published before 2018
		Studies with data collected before 2018
Setting	High‐income countries Organisation for Economic Co‐operation and Development (OECD) ‐ https://data.worldbank.org/?locations=XD-OE	Non‐high‐income countries
Language	English	
Peer reviewed	Papers must be peer reviewed	Papers that are not peer reviewed

Other factors considered in inclusion and exclusion criteria included study type and study period. Grey literature was included due to the recent study period; it is acknowledged that the cost‐of‐living crisis is still an ongoing situation. As such relevant manuscripts which were close to publication were sought after through contacting researchers in the relevant fields, through relevant mailing lists. The chosen study period was from 2018 onwards, as this includes BREXIT (in the UK), the COVID‐19 pandemic and the concurrent cost‐of‐living crisis which many high‐income countries are experiencing. This also considers the long‐term austerity measures populations in high‐income countries have experienced. Although the time periods of previous reviews overlap with the current review, the current timeline is more recent.

The search strategy was developed through the recommended stages of the JBI scoping review manual (Peters et al. 2024) and consultation with a research librarian. The search terms first tried in the CINAHL database were infant OR child* AND ‘food insecur*’ OR ‘food poverty’ AND household OR family OR home, with date specified as part of search to reflect inclusion criteria. This highlighted the need to be more specific and refine the terms to produce more appropriate studies. Relevant papers were found and the words and phrases they used noted to help produce a relevant and refined search. The final search terms (see Table 2) were used in all four databases—CINAHL, PubMed, Web of Science and Scopus—see Appendix for the full list. The search was carried out on 8 April 2024 (by G.H.), and electronic database alerts were created to identify any newly published papers following this.

**Table 2:** Search terms used in article search.

Search number	PEO component	Search terms
1	Population	Child*, Toddler*, Baby, Babies, Infan*
2	Exposure	“Food insecur*” household, “Food poverty”, “Food secur*” household, Food insufficien*, Food depriv*
3	Outcomes	Nutrition*, Diet*, “feeding practices”, responsive feeding, “complementary feeding”

### 2.3 Reference Management

References were exported into the referencing software Zotero (https://www.zotero.org/). Then references were imported into Covidence (https://www.covidence.org), a web‐based review management software, designed to aid in systematic and scoping reviews, where duplicate studies were removed automatically and manually. Covidence ensures the paper review process is rigorous and aids in minimising bias from reviewers by moving articles through the stages (title and abstract screen, then full text screen) according to reviewer's decisions without reviewer contact to maintain independence. An additional benefit is it helps organise the articles and saves the reviewers progress so they can easily identify where they are in the process if they need to pause and then return.

### 2.4 Study Selection

Before screening, reviewers (G.H. and L.S.) discussed the title and abstract screening process, including software navigation and inclusion and exclusion criteria. For every title and abstract, both reviewers voted ‘yes’, ‘no’, or ‘maybe’. If voted ‘yes’ or ‘maybe’, the article progressed to the next stage. If contradicting votes occurred, articles moved to the ‘resolve conflicts’ section, where the original vote is concealed to reduce bias. Articles in this section were discussed between the two reviewers (G.H. and L.S.), until a unanimous vote was agreed. The next stage was the full text review. At this stage reviewers could either include or exclude the article. Excluded articles required a justification from the reviewer. Covidence provides a pre‐specified list of reasons which were edited by the primary reviewer (G.H.) to make more relevant to the current review. Again, conflicting votes were discussed, until a unanimous vote was agreed. A third reviewer (L.W.) was available if a unanimous vote was not agreed, however, they were not required as a decision was always agreed between the two reviewers.

### 2.5 Charting the Data

Components of the data extraction tables were discussed by the reviewers (G.H. and L.S.) to ensure study characteristics of interest were included. These were citation details, country of origin, study aims and design, definition of household food insecurity used, recruitment method, infant's/child's age and gender, parent gender, tools used to measure outcomes, and key findings.

Quality analysis was conducted, by the primary reviewer, to assess the methodology of the studies to help inform future research, not to quality assess for potential bias in the studies. The Mixed Methods Appraisal Tool version 2018 (Hong et al. 2018) was used as this review included both qualitative, quantitative and mixed methods studies in inclusion criteria, and this tool was created to appraise all of these. This consists of two screening questions, before asking five questions to assess the methodological quality of the study, which vary based on the study methodology (Hong et al. 2018). Overall scores are not calculated, instead it is assessed as to whether each of the five criteria is met or not, with response options ‘yes’, ‘no’ and ‘cannot tell’.

### 2.6 Collating the Results

The extracted data was exported from Covidence into a Microsoft Excel spreadsheet. A narrative synthesis was conducted on the data to highlight the similarities and differences in the final studies, to understand how food insecurity impacts diet and feeding practices, what is already known in the literature, and where future research is needed. Initial themes were created by the primary author and then reviewed and refined (L.W., S.K. and A.B.).

### 2.7 Ethics Statement

This systematic scoping review analyses existing published literature and does not involve the collection of primary data, therefore, ethical approval was not required. However, all research practices adhered to the principles of academic integrity, including proper citation of sources and transparent reporting of findings.

## 3 Findings

### 3.1 Literature Search

**Figure 1:**
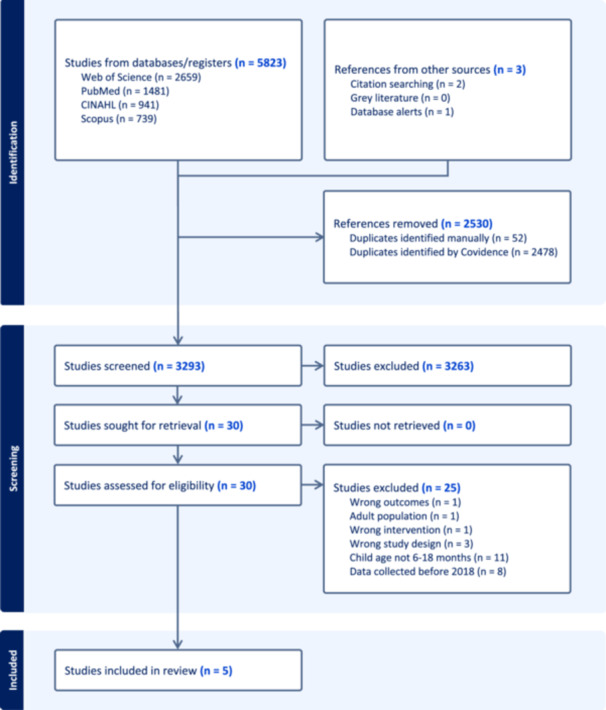
PRISMA‐ScR flow diagram.

Across the four databases, citation searches, and email alerts, 5822 articles were imported into Covidence, duplicates were removed so that 3293 titles and abstracts were screened from which 30 articles full texts were screened. Reasons for excluding articles at full‐text stage are reported using a PRISMA‐ScR flow diagram (see Figure 1). The final review included five articles.

### 3.2 Study Characteristics

The included articles were published from 2020 to 2024, with 80% of them published in 2024 (see Table 3). There were only three countries of origin where data was collected, Australia (*n* = 3), the United States of America (*n* = 1) and New Zealand (*n* = 1). All quantitative studies were cross‐sectional cohort studies, and all measured household food security status. Both qualitative studies looked at both diet and feeding practices. See the Supporting Materials for the full characteristics of included studies.

**Table 3:** General characteristics of studies included.

Study characteristics (*n* = 5)	*n*	%
Year of publication		
2020	1	20
2024	4	80
Country of origin		
Australia	3	60
America	1	20
New Zealand	1	20
Study design		
Qualitative	2	40
Quantitative ‐ cross sectional	3	60
Household food security status measured?		
Yes	3	60
No	2	40
Outcomes		
Diet	1	20
Feeding practices	1	20
Diet and feeding practices	3	60

#### 3.2.1 Household Food Insecurity

Different definitions of household food insecurity were used, but with similar elements, covering the different dimensions of household food insecurity—quantity and quality of food and access to food. However, only two definitions mentioned meeting dietary needs and preferences or acceptable foods, which were from the American Dietetic Association (Olson and Holben 2002) and a paper by Anderson (1990).

In total, four studies focused on household food insecurity. Three studies used tools to measure household food security status. Fernández et al. (2020) used one dichotomous tool, the two‐item Hunger Vital Sign survey (Hager et al. 2010). Katiforis et al. (2025) used the validated eight‐item food security measurement tool for New Zealand households, which categorises into three levels of food insecurity—food secure, moderately food insecure and severely food insecure (Parnell and Gray 2014). So et al. (2024a) used two tools to compare reliability: the dichotomous question from the Australian National Health Survey (ANHS) and the 18‐item United States Department of Agriculture Household Food Security Survey Module (HFSSM) (Australian Bureau of Statistics 2015; USDA 2024). The results showed a stark difference, 37% of households were food insecure according to the ANHS but with the HFSSM (dichotomised into food secure or insecure) 77% of households were food insecure (So et al. 2024a). The categorised data (split into severity of food insecurity) from the HFSSM was grouped into adult and child food security status, as the 18‐item HFSSM has eight‐items for child experience, this was the only paper to do this and highlight the child's experience of food security status can differ from the adults (So et al. 2024a).

Both qualitative studies used financial hardship as a proxy measure, without measuring household food security status directly. For both studies, parents were eligible if they self‐identified as experiencing financial hardship, which was measured through a single question (Baxter et al. 2024; So et al. 2024). So et al. (2024) observed some fathers had experienced acute or chronic food insecurity and food insecurity was also included as part of a theme. Baxter et al. (2024) mentioned food insecurity in the introduction and provided a definition, but food insecurity was not in the aim or created as a theme in itself, it was recognised as a code when forming themes. Different dimensions of food insecurity were mentioned throughout the results, such as quantity and quality of food, and the worry of running out of food (Baxter et al. 2024).

### 3.3 Participant Characteristics

Across the included studies there were 1044 parent/child dyads participating, with sample size ranging from 25 to 604. Child sex was only collected in three of the studies (see Table 4). Parent sex was collected across four studies, and one study reported 99% of participants were mothers. Two of the studies explored fathers perspectives, and one study included one father, therefore an eighth of parents are male (Baxter et al. 2024; So et al. 2024; So et al. 2024a). Child age is discussed below, in its own Section, 3.3.1.

**Table 4:** Child and parent sex.

	Male		Female		Not reported		Total
	*n*	%	*n*	%	*n*	%	*n*
Child	534	51.1	451	43.2	59	5.7	1044
Parent	131	12.5	905	86.7	8	0.8	1044

#### 3.3.1 Child Age

Due to the individual studies' age ranges, the actual ages of the participants differ slightly from the set study inclusion criteria for this review. Katiforis et al. (2025) set the narrowest age range criteria of 7–9.9 months old, the mean age of participants was 8.4 (standard deviation = 0.8). Next, Fernández et al. (2020) set the criteria of under 2 years old, the median age of participants was 6 months old. Baxter et al. (2024) set the age range 6 months to 3 years for the index child, the age range of all children of families included in the study was given, 7 months to 13 years, as demographic data was collected for all children in the household. So et al. (2024a) set the age range 6 months to 5 years, but participants ranged from 5 months to 23 months, extending the criteria below the initial range, as two infants had started solids before 6 months old. So et al. (2024b) also set the age range 6 months to 5 years, but only provided the median age of participants 2.9 years (interquartile range 1.3–4.8 years).

### 3.4 Study Themes

From the narrative synthesis of the data, the themes of diet and feeding practices were considered to answer the research question, with subthemes constructed within them. Diet quality was the main aspect of the diet impacted. In terms of feeding practices, nonresponsive or coercive practices were more likely followed. Additionally, regarding complementary feeding approach, there was significant difference in feeding practices at 6 months but there was no significant difference by 8 months old. For both diet and feeding practices, the reasons given by parents for the changes were to prevent hunger and food waste.

#### 3.4.1 Diet

Studies aiming to understand parent's experience of feeding their child when experiencing financial hardship found diet quality was impacted by household food insecurity in multiple ways. Parents reported decreasing purchasing of disliked foods, only buying foods which had previously been accepted by their child (Baxter et al. 2024; Katiforis et al. 2025; So et al. 2024). Food banks were utilised to protect child diet (Baxter et al. 2024), but it was also reported that food bank parcels reduced variety of foods (So et al. 2024). Alongside decreasing certain foods, parents reported increasing purchases of perceived high satiety foods to make more filling meals and help stretch meal portions further (Baxter et al. 2024; So et al. 2024). In several studies, it was either reported, or authors surmised that parents prioritised their child's food intake over their own, with parents restricting their intake by skipping meals or giving the more nutritious foods to their child (Baxter et al. 2024; So et al. 2024; So et al. 2024a). Additionally, parents emphasised providing home‐cooked meals to establish healthy eating habits and trying to prioritise fruit and vegetables (Baxter et al. 2024; So et al. 2024).

The other two studies focusing on infant diet explored use of specific food and drink products, finding infants experiencing food insecurity were more likely to consume the products which have high sugar content (Fernández et al. 2020; Katiforis et al. 2025). Fernández et al.'s (2020) quantitative cross‐sectional cohort study compared sugar sweetened beverage intake of food secure and food insecure households. Participants were all low‐income mothers registered onto The Special Supplemental Nutrition Programme for Women, Infants, and Children (WIC) (Fernández et al. 2020). In this study's sample, nearly a third of infants experiencing food insecurity habitually consumed sugar sweetened beverages and were twice as likely to consume sugar sweetened beverages as their food secure counterparts (Fernández et al. 2020). Any consumption was considered habitual by the authors as recommendations outline infants should not be consuming any added sugars (Fernández et al. 2020).

Katiforis et al.'s (2025) cross‐sectional cohort study compared baby food pouch use across three household food security statuses (food secure, moderately food insecure and severely food insecure). In this study's sample, nearly half of 6‐month‐olds experiencing severe food insecurity were frequently fed baby food pouches compared to much lower proportions in the other groups (Katiforis et al. 2025). Food secure 6‐month‐olds were least likely to be fed baby food pouches frequently (Katiforis et al. 2025). The authors defined frequent pouch use as anything from more than once a day to 5‐6 times a week (Katiforis et al. 2025). This trend continued when the infants were older. At the current age of the study (mean 8.4 months), nearly two‐thirds of infants experiencing severe food insecurity were frequently fed baby food pouches (Katiforis et al. 2025). Severely food insecure mothers were five times more likely to frequently use baby food pouches than food secure mothers (Katiforis et al. 2025). A high percentage of mothers experiencing severe food insecurity reported pouches as an easy way to introduce fruit, vegetables and meat (Katiforis et al. 2025).

#### 3.4.2 Feeding Practices

It was found that feeding practices were impacted by household food insecurity. So et al. (2024a) found food insecurity is associated with coercive practices such as persuasive feeding, in fathers with children under 2 years old. Moreover, parents reported coercive practices, such as encouraging extra bites, due to food waste concerns, and in reaction to fussy eating, mealtime tantrums, and appetite fluctuation (So et al. 2024b). Concerns of children under‐eating prompted fathers to use more pressuring and monitoring behaviours such as using rewards, bribery and setting punishments (So et al. 2024b). Fathers also reported nonresponsive feeding practices such as using electronic devices to keep children at the table and create a calm atmosphere and placing restrictions on what and how much food children could eat due to financial hardship (So et al. 2024b). Several studies stated food insecurity was a barrier to responsive feeding practices (Baxter et al. 2024; So et al. 2024b; So et al. 2024a).

On the other hand, some fathers were aware of their worries of food waste and consciously tried to not action them, for example resisting the thought that their child needs to finish their plate (So et al. 2024). Moreover, several parents recounted how important regular routine mealtimes are, sitting and eating as a family, for children to learn healthy eating habits and social skills (Baxter et al. 2024; So et al. 2024).

Only one study explored the impact of household food insecurity on complementary feeding approach, finding there was initially a significant impact (Katiforis et al. 2025). Mothers experiencing severe food insecurity were significantly less likely to be following the full baby‐led weaning (BLW) approach at 6 months old compared to those experiencing moderate food insecurity and food security; but there was no significant difference between groups at 8 months old (Katiforis et al. 2025). The percentage of mothers using the traditional spoon‐feeding approach at 6 months old was comparable between the three food security status groups (Katiforis et al. 2025).

#### 3.4.3 Parental Worries

In four studies, parents reported or authors surmised that purchasing decisions, meals provided and feeding practices were all altered to ensure children did not go hungry or due to concerns about food waste (Baxter et al. 2024; Katiforis et al. 2025; So et al. 2024; So et al. 2024a). Aiming to prevent hunger and food waste was at the root of the adaptations and practices. Parents main concern was food waste, as they perceived this as a waste of money (Baxter et al. 2024; So et al. 2024). One parent reported that this caused tension between her partner and herself as he was the main earner of the household, but she wanted to allow their child to explore and play with food which created food waste as food is not always eaten (Baxter et al. 2024). Two studies also mentioned the increased mental load this applied to parents; the planning of where to access food, which foods to purchase, meals to prepare, as well as the worry of food waste and child hunger (Baxter et al. 2024; So et al. 2024) Table 5.

**Table 5:** Study themes and sub‐themes.

Study	Theme	Sub‐theme
Baxter et al. (2024)	Diet, feeding practices, parental worries	Diet quality. Food waste. Hungry children
Fernández et al. (2020)	Diet	Diet quality
So et al. (2024)	Diet, feeding practices, parental worries	Diet quality. Food waste. Hungry children
So et al. (2024a)	Feeding practices. Parental worries	
Katiforis et al. (2024)	Diet, feeding practices	Baby food pouches. Complementary feeding approach

### 3.5 Quality Appraisal

All articles had clear research questions, and the data addressed the research questions. Tables 6 and 7 outline if the studies met the methodological criteria.

**Table 6:** Quality appraisal summary of two qualitative studies.

	Meet the criteria? (yes, no, cannot tell)	
MMAT question criteria	Baxter et al. (2024)	So et al. (2024b)
1. Is the qualitative approach appropriate to answer the research question?	Yes	Yes
2. Are the qualitative data collection methods adequate to address the research question?	Yes	Yes
3. Are the findings adequately derived from the data?	Yes	Yes
4. Is the interpretation of results sufficiently substantiated by data?	Yes	Yes
5. Is there coherence between qualitative data sources, collection, analysis and interpretation?	Yes	Yes

**Table 7:** Quality appraisal summary of two quantitative non‐randomised studies.

MMAT question criteria	Meet the criteria? (yes, no, cannot tell)		
	Fernández et al. (2020)	So et al. (2024a)	Katiforis et al. (2024)
1. Are the participants representative of the target population?	Yes	Yes; although mostly biological fathers residing with child	No; although selection bias acknowledged
2. Are measurements appropriate regarding both the outcome and intervention (or exposure)?	Yes	Yes	Yes
3. Are there complete outcome data?	Yes	No; missing data for the food security measure	Yes; all participants had complete food security measure
4. Are the confounders accounted for in the design and analysis?	Yes	Yes	Yes
5. During the study period, is the intervention administered (or exposure occurred) as intended?	Yes	Yes	Yes

## 4 Discussion

This systematic scoping review explored how household food insecurity impacts diet and feeding practices during the complementary feeding period. Five studies were reviewed that considered these two outcomes. Three themes were constructed: diet, feeding practices and parental worries. The quality of each of the studies was also assessed.

Infant diet was explored in four studies. Only two studies measured the relationship between food insecurity and diet, but looked at intake of a specific food or drink item (Fernández et al. 2020; Katiforis et al. 2025). However, none of the included studies measured the whole of infant's diet, such as food and drink intake using a food frequency questionnaire. This would highlight the impact on different aspects of diet such as how different food groups are impacted, quantity of food, and variety of food. In regard to complementary feeding, this is important as recommendations outline to give fruit and vegetables to begin with, and then protein and carbohydrates, avoiding high fat and sugar foods until 2 years old (World Health Organisation 2023).

Additionally, only one of the studies explored shop bought baby food product use, specifically pouches, and found food insecure mothers are more likely to buy baby food pouches (Katiforis et al. 2025). This is a relatively new area of research; however, the most recent statistics and literature show mothers living in high‐income countries, with low socioeconomic status are more likely to introduce solid foods early (Barrera et al. 2018; Harrison et al. 2017; Helle et al. 2018; McAndrew et al. 2010). When solid foods are introduced before the infant is developmentally ready, purees are usually required, making it more likely for baby food products to be used (Alpers et al. 2019). Recent studies indicate parents reasons for using baby food products include convenience, time saving, and infant's perceived enjoyment of products (Hollinrake et al. 2024; Isaacs et al. 2022). Similarly, the study included in this review found these same reasons for use, and also noted food insecure mothers perceived baby food pouches as nutritious (Katiforis et al. 2025). Concern about baby food products is due to the high sugar content, and pouches add the concern of infants sucking the puree out rather than being spoon fed (Dunford et al. 2024; Hutchinson et al. 2021; Westland and Crawley 2018). Although, one study found frequent pouch use was not associated with weight or energy intake but was associated with higher food fussiness and selective eating (Cox et al. 2024). There is a clear gap of investigating the relationship between household food security status and use of baby food products and or complementary feeding approach, in more high‐income countries. Future research could explore this relationship, building on the current literature, and findings from New Zealand (Katiforis et al. 2025).

Some studies reported parents prioritised their child's diet by either giving them the healthiest foods or sacrificing their diet, fathers also mentioned wanting to form healthy eating habits for their child through homecooked meals (Baxter et al. 2024; So et al. 2024). Additionally, the WIC programme is known to provide nutrition information to parents, and all mothers in Fernández et al.'s (2020) study were enroled in WIC, yet there was a difference between food secure and food insecure infants sugar‐sweetened beverage intake. This implies it is not lack of knowledge or awareness of healthy diets, but it is the lack of access and ability to afford a healthy diet, and potentially many other wider factors, such as family influence, that prevents food insecure households from following healthy eating guidelines and recommendations. Many interventions focus on improving knowledge, the focus should be to influence policy to enable individuals to have the choice of healthy foods, through improving access and affordability.

Parental feeding practices were considered in three studies. Food insecurity was found to be a barrier to responsive feeding practices, and increased chance of coercive feeding practices (Baxter et al. 2024; So et al. 2024b; So et al. 2024a). However, the association between household food security status and feeding practices was only considered by proxy of household chaos (So et al. 2024a). Although, a study discovered young children who were avid eaters (defined as higher levels of food responsiveness, enjoyment of food, and emotional over‐eating combined with lower satiety responsiveness, slowness in eating and food fussiness) were found to have experienced greater food insecurity than three other eating profiles (Pickard et al. 2023). Responsive feeding helps infants to learn their hunger and fullness signals, and to regulate their food intake in response (Brown and Lee 2015). Eating behaviours learnt in infancy can then track into childhood and adulthood, impacting future eating behaviours, dietary patterns and weight trajectory (Costa and Oliveira 2023; Dubois et al. 2022). The included studies in this review showed an increase in use of coercive feeding practices, when experiencing food insecurity, decreasing responsive feeding practices. Therefore, infants experiencing food insecurity may be less likely to be taught satiety responsiveness which may impact them across their life span.

The only study focusing on the complementary feeding period somewhat agreed with the other studies looking at feeding practices, at 6 months old infants experiencing severe food insecurity were less likely to be baby‐led weaned than the more food secure infants (Katiforis et al. 2025). This means infants experiencing severe food insecurity were less likely to be satiety responsive as baby‐led weaning is associated with promoting satiety responsiveness (Brown and Lee 2015). Additionally, if baby‐led weaning is followed, baby food products are less likely to be used, as the focus is on giving solid foods. However, by 8 months old there was no significant difference in complementary feeding approach between household food security status (Katiforis et al. 2025). Future research could investigate the effect food insecurity has on the ability for parents to follow responsive feeding practices in the complementary feeding period, as this is another gap which has been identified in this review.

Mealtime routine and family mealtimes were also considered within feeding practices. Some parents acknowledged their importance for learning healthy eating habits, however others reported using strategies to encourage their child to eat such as food and non‐food rewards (Baxter et al. 2024; So et al. 2024b). Parents eating with their children is important as it is an opportunity for modelling and for parents to support their child trying new foods. Research with young children (2–4 years old) found parental use of modelling and physical prompting encouraged children to try novel fruit, in food responsive children, and children with low food responsiveness responded best to parental food modelling without physical prompts (Blissett et al. 2016). Parents using rewards also increases the chance of young children (2–4 years old) trying novel fruits, however it was also found to be associated with children's refusal (Blissett et al. 2012). This shows that overall, similar feeding practices will support children to try new foods, but some feeding practices, such as using rewards, can have both the desired and undesired outcomes (Blissett et al. 2012, 2016). Hence, food modelling and prompting, through eating together can encourage children to try new foods, increasing their variety of foods (Blissett et al. 2012, 2016). Therefore, further research may look at the impact of household food insecurity on the ability to eat meals together, and the opportunity for modelling, and children's acceptance of new foods in the complementary feeding period, when new foods are given regularly. This would be of interest as this review highlights parents are concerned about food waste, and therefore new food acceptance is important from a financial viewpoint. Although, it must also be noted, reduced variety of foods was also linked with food insecurity so there may be less opportunities for new foods to be tried if a household is food insecure.

Many parents reported fear of food waste as that equated with a waste of money (Baxter et al. 2024; So et al. 2024b). This led one parent to recount increased tension in the household as they wanted to allow their child to play with food however their partner saw this as wasting food as it was not being eaten (Baxter et al. 2024). During complementary feeding and into early childhood, playing with food encourages exploring textures and learning about food. Transitioning from milk only to different foods with different textures is an important element of complementary feeding as it helps inform children and prepare them for differentiation in foods throughout life (Ventura and Worobey 2013). There is a gap in the literature, exploring if there is a relationship between household food security status, fear of food waste, and allowing children to play with and explore food. There could be a knock‐on effect of children then more likely being fussy eaters or having a smaller variety of foods they will eat into childhood, if they have not been able to have this learning experiences. Although, it must be considered, in some cultures, playing with food is considered disrespectful or taboo, making this a complex and culturally sensitive issue to investigate.

Child age was a specific inclusion criterion in this review, as the complementary feeding period is from around 6–18‐months old. The included studies inclusion criterion and participant demographics all varied on this, with all but one being a broader range than this reviews criterion. Previous studies have investigated the association at different ages such as at 2 months old (Orr et al. 2019), this could not be included as the participants were too young, and 10 months old (Gross et al. 2018), this could not be included as the data was collected before 2018. This highlights an interest in the literature of how food insecurity impacts infant feeding, showing it is not a completely novel topic. Although, this is a current knowledge gap, future research may investigate this age group, and nutritional stage, to gain an understanding of the impact household food security status has on the complementary feeding period in the current cost‐of‐living crisis, in high‐income countries. This would add to the literature, as households are currently experiencing deeper poverty than we have seen previously (JRF 2024), and more households are food insecure than before (The Trussell Trust 2023), with different demographics who would not have previously been considered to experience food insecurity; as households on higher incomes are now self‐reporting being food insecure (Thomas et al. 2022).

Household food security status was either measured directly, in quantitative studies, or by proxy of financial hardship, in qualitative studies. Although valid tools were used when measured, these come with limitations. Short item surveys only produce dichotomous results, unable to show severity of the food insecurity, such as the 2‐item Hunger Vital Sign survey, which the study recognised as a limitation (Fernández et al. 2020). Whereas the advantage of the longer item surveys such as the Household Food Security Survey Module (HFSSM) is that it categorises food security status into four categories, which was recognised in So et al. (2024a). However, a longer item survey comes with a higher participant burden. A limitation of this is there is not one tool that is universally used, therefore it is challenging to compare between studies, as the food security status may not be comparable. As shown in one of the review studies, two measures used presented different percentages of household food insecurity (37% for the short survey and 77% for the HFSSM) in the same sample (So et al. 2024a). Hence, researchers focusing on food insecurity may need to agree on a measure which is suited to all populations, without being too burdensome on participants, that has high validity, so that results can be compared and also be more generalisable. Although, it must be noted this is a lot of requirements for one tool. Alternatively, there needs to be repetition and replication of studies, using different tools, to gain a better understanding of how many households are experiencing food insecurity during complementary feeding.

An advantage of quantitative research and measuring household food security status is the groups, whether dichotomised or categorised, can then be compared to see if there is a difference in outcomes, which if there is, strengthens any associations. Measuring indirectly through self‐reported financial hardship, may be better suited to the qualitative nature of studies, however, not using a specific measure is likely to affect the experience of food insecurity captured. For example, without a tool you cannot measure the severity of food insecurity experienced, and may not capture a wider variety of participants who are more financially secure but food insecure, a group being increasingly observed (Thomas et al. 2022). However, the qualitative approach helps to understand the lived experience of individuals, and highlight areas not yet known or understood. Hence, future research would be beneficial in qualitative and quantitative approaches or using a mixed methods approach. Additionally, another gap identified which could be focused on, is to invite all parents to participate, and then measure household food security status, as Katiforis et al. (2025) did, to capture those who are financially secure but food insecure.

The characteristics of the reviewed studies were largely similar. Of the five studies, there were only three countries of origin, none of those being the UK. This highlights a gap that research is needed in the UK, to understand how the cost‐of‐living crisis has impacted complementary feeding. Arguably, the countries are high‐income countries, and the results can be somewhat generalised to the UK; however, different countries have different policies, welfare regimes and public services available. Even within a country there are different local community services and support. Moreover, three of the reviewed studies came from the same research team, which indicates the need for repetition and replication of these study designs in different locations with different cultures and welfare regimes. Therefore, caution must be taken when using the results found in this review to predict outcomes and provide support in other high‐income countries, as they cannot necessarily be generalised. Research in other high‐income countries, such as the UK, replicating the studies in this review, would add to the literature on this topic.

Unusually, two studies focused on father's experiences of feeding and household food insecurity (So et al. 2024b; So et al. 2024a). This is positive as fathers are often a population that are less seen in infant nutrition research (Davison et al. 2016). However, mothers usually hold most of the mental load and complete most of the food work so are more aware of these circumstances (O'Connell and Brannen 2016). A study which was excluded from this review due to children being older than the inclusion criteria explored mother/father/child triads and asked both parents to complete a household food security status questionnaire separately (Foster et al. 2019). Fathers were found to be food secure on average, whereas mothers were marginally food secure on average, indicating a different perception of household food security status depending on who was asked (Foster et al. 2019). It must be considered this mother/father dynamic can only be applied to a nuclear family, and with a lot of households breaking this mould, and breaking gender stereotypes, or indeed other adults caring for children such as grandparents. Therefore, the adult responsible for most of the food work and feeding infant is the best person to reach out to, to gain the most accurate information about household food security status. Hence, future research should consider recruiting the primary food worker of the household with an infant, and collecting data from them, which may give a more accurate picture of household food insecurity, and the food management and work required.

In summary, the research gaps identified in this review are as follows:

- Measuring the impact of household food insecurity on infant diet as a whole.
- More studies, measuring the impact of household food insecurity on baby food product use and complementary feeding approach.
- The impact of household food insecurity on responsive feeding during complementary feeding.
- The impact of household food insecurity on the ability to eat meals together, and the opportunity for modelling, and children's acceptance of new foods in the complementary feeding period.
- The relationship between household food security status, fear of food waste, and allowing children to play with and explore food.
- The impact household food security status has on the complementary feeding period in the current cost‐of‐living crisis, in high‐income countries.
- Refining a tool to be used by all research on this topic to compare and contrast between studies and have a comparable understanding of household food insecurity, or repetition and replication of studies in different high‐income countries.

### 4.1 Implications for Research and Policy

Many knowledge gaps have been identified from this review. It is important that future research addresses them as if household food insecurity is having a detrimental impact on complementary feeding that could impact a proportion of the next generation for their lifetime. The first 1001 days is a time of rapid growth and development, if infants are not meeting their dietary requirements this will impact these. Research can help change policy to ensure infant diet and health is considered, and financial support provided to parents and carers to help them provide a diet to meet guidelines, without finances causing purchasing restrictions. This support would not only help parents and carers in the current cost‐of‐living crisis but continuing into the future too.

### 4.2 Strengths and Limitations

This systematic scoping review provided a thorough exploration of how household food insecurity impacts complementary feeding, specifically diet and feeding practices, in high‐income countries. Moreover, high reviewer agreement was achieved throughout the screening stages due to rigorous discussions between reviewers before commencing to ensure inclusion and exclusion criteria were clearly understood. Additionally, methodological quality was maintained by continually referring to the PRISMA extension for scoping review checklist (Tricco et al. 2018).

We must also acknowledge the limitations of this systematic scoping review. The inclusion and exclusion criteria could be considered too narrow and were at times difficult to apply, as such some studies were included in the review which did not fully meet inclusion criteria, to avoid excluding all studies. For example, data was collected in June 2017 in Fernández et al. (2020). Additionally, the time period of this review overlapped slightly with previous reviews on similar topics (Baxter et al. 2022; Bell et al. 2023), however, this was a more recent timeline with a more specific focus, identifying different and more recent studies. Moreover, it is likely there are other papers which explore household food insecurity and the impact on complementary feeding which did not use specific terms in titles and abstracts or through human error were excluded. Furthermore, the PEO framework used may not have been the most appropriate. The authors acknowledge the PCC (Population Concept Context) framework may have been a better fit for this scoping review (Peters et al. 2024).

## 5 Conclusion

This systematic scoping review aimed to understand how household food insecurity affects complementary feeding. The findings suggest that diet and feedings practices could be negatively affected by household food insecurity. Diet quality and variety may reduce, baby food pouches may be more likely to be used, and persuasive feeding practices could more likely be used due to fear of food waste and child hunger. Moreover, this reduces the opportunity to try new tastes and textures and learn from these experiences. Additionally, it hinders the chance to learn satiety responsiveness, an important eating behaviour to learn at a young age. There was limited evidence in this review, across high‐income countries. The studies only came from three countries of origin and only one investigated the complementary feeding period specifically. Moreover, overall diet was not measured, and in two studies household food security was measured indirectly through financial hardship. Due to these knowledge gaps, the effect of household food insecurity, in other high‐income countries, on complementary feeding is uncertain, and cannot be assumed. However, if future research addressed this uncertainty, it could help inform policy and increase support for families with infants and young children, living in other high‐income countries, such as the UK, in the current cost‐of‐living crisis and beyond.

## Author Contributions

Grace Hollinrake, Laura L. Wilkinson, Sophia Komninou and Amy Brown conceptualised the research. Grace Hollinrake and Lowri Stevenson performed the research. Grace Hollinrake analysed the data. Grace Hollinrake, Laura L. Wilkinson, Sophia Komninou and Amy Brown wrote the paper.

## Conflicts of Interest

The authors declare no conflicts of interest.

## Supporting information

Data_extraction_table.

## Data Availability

The data that support the findings of this study are available from the corresponding author upon reasonable request.

## Search terms

**CINAHL**

TI (child* OR toddler* OR baby OR babies OR infan*) OR AB (child* OR toddler* OR baby OR babies OR infan*) OR (MH “Infant + ”) OR (MH “Child, Preschool”).

AND TI (Food insecur* N2 household OR “Food insecur*” OR “Food poverty” OR Food secur* N2 household OR “food secur*” OR food N2 insufficien* OR food N2 depriv*) OR AB (Food insecur* N2 household OR “Food insecur*” OR “Food poverty” OR Food secur* N2 household OR “food secur*” OR food N2 insufficien* OR food N2 depriv*) OR (MH “Food Security + ”).

AND TI (nutrition* OR diet* OR “feeding practices” OR responsive feeding OR “complementary feeding”) OR AB (nutrition* OR diet* OR “feeding practices” OR responsive feeding OR “complementary feeding”) OR (MH “Infant Nutrition + ”) OR (MH “Infant Nutritional Physiology + ”).

Limits 2018‐current, English language.

**SCOPUS**

TITLE‐ABS‐KEY (child* OR toddler* OR baby OR babies OR infan*)

AND TITLE‐ABS‐KEY ((“food insecur*”) OR (food insecur* W/2 household) OR (“food poverty”) OR (“food secur*”) OR (food secur* W/2 household) OR (food W/2 insufficien*) OR (food W/2 depriv*)).

AND TITLE‐ABS‐KEY (nutrition* OR diet* OR “feeding practices” OR responsive feeding OR “complementary feeding”).

Limits 2018‐current, English language.

**Web of science**

Topic = (child* OR toddler* OR baby OR babies OR infan*).

AND Topic = (“food insecur*” OR food insecur* NEAR/2 household OR “food secur*” OR food secur* NEAR/2 household OR “food poverty” OR “food depriv*” OR “food insufficien*”).

AND Topic = (nutrition* OR diet* OR “feeding practices” OR responsive feeding OR “complementary feeding”).

Limits 2018‐present, English language.

**PubMed**

child*[Title/Abstract] OR toddler[Title/Abstract] OR baby[Title/Abstract] OR babies[Title/Abstract] OR infan*[Title/Abstract].

AND (“food insecur*”[Title/Abstract] OR “food secur*”[Title/Abstract] OR “food poverty”[Title/Abstract] OR “food depriv*”[Title/Abstract] OR “food insufficien*”[Title/Abstract]).

AND (nutrition*[Title/Abstract] OR diet*[Title/Abstract] OR “feeding practices”[Title/Abstract] OR responsive feeding[Title/Abstract] OR “complementary feeding”[Title/Abstract]).

Limits 2018‐present, English language.
