# Supplementary material for: How Does Household Food Insecurity Impact Complementary Feeding, in High Income Countries, in a Cost‐of‐Living Crisis? A Systematic Scoping Review
Source: Matern Child Nutr. 2025 Aug 20;22(1):e70082. doi: 10.1111/mcn.70082 (PMC12893513; doi:10.1111/mcn.70082)
Supplement: Supplementary file 1 — Data_extraction_table. [file MCN-22-e70082-s001.docx]

**Data Extraction table**

| **Study ID** | **Baxter 2024** | **So 2024** | **FernÃndez 2020** | **So 2024** | **Katiforis et al., 2024** |
| --- | --- | --- | --- | --- | --- |
| **Title** | Food Insecurity and Feeding Experiences Among Parents of Young Children in Australia: An Exploratory Qualitative Study | "You just have to spread it thin":  Perceptions and feeding experiences of Australian fathers of young children living with disadvantage | Food Insecurity and Sugar-Sweetened Beverage Consumption Among WIC-Enrolled Families in the First 1,000 Days | Dads at Mealtimes: Associations  between Food Security, Household and Work Chaos, and Paternal Feeding Practices among Australian Fathers Living with Disadvantage | Household food insecurity and novel  complementary feeding methods in New Zealand families |
| **Authors** | Baxter, K.A.; Nambiar, S.; Penny, R.; Gallegos, D.; Byrne, R. | So, J.T.H., Byrne, R., Nambiar, S., Gallegos, D., Baxter K.A. | Fernandez, C.R.; Chen, L.; Cheng, E.R.; Charles, N.; Meyer, D.; Monk, C.; Baidal, J.W. | So, J.T.H.; Nambiar,  S.; Byrne, R.; Gallegos, D.; Baxter, K.A. | Katiforis, I., Smith, C., Haszard, J.J., Styles, S.E., Leong, C., Taylor, R.W., Conlon, C.A., Beck, K.L., von Hurst, P.R., Te Morenga, L.A., Daniels, L., Brown, K.J., Rowan, M., Casale, M., McLean, N.H., Cox, A.M., Jones, E.A., Bruckner, B.R., Jupiterwala, R., Wei, A., Heath, A-L, M. |
| **Year** | 2024 | 2024 | 2020 | 2024 | 2024 |
| **Country** | Australia | Australia | United States | Australia | New Zealand |
| **Aim of study** | What are the experiences and approaches of parents facing financial hardship in feeding their children, and how might this inform the development of child feeding programs? | This qualitative study aimed to explore fathers:  (i) contributions and perceived roles in child eating and feeding, and (ii) perspectives of enablers and barriers for responsive feeding practices and paternal involvement, within the context of socioeconomic disadvantage and food insecurity | The objectives of this study were  to examine the association between household food insecurity and habitual SSB consumption in mothers and infants during the first 1,000 days. | This study aimed to achieve three objectives among Australian fathers:  1) describe paternal involvement in child eating,  2) assess the prevalence and severity of food insecurity while exploring its relationship with household and work chaos,  3) identify paternal feeding practices and examine how these practices are associated with household food insecurity, household and work chaos, and other sociodemographic factors. | The aim of this study was to investigate associations  between household food insecurity and both commercial baby food pouch use and BLW. |
| **Study design** | Qualitative research | Qualitative research | Cross sectional study | Cross sectional study | Cross sectional study |
| **Start date** | Aug-21 | Apr-22 | Mar-17 | Mar-22 | Jul-20 |
| **End date** | Jan-22 | Sep-22 | Jun-17 | Sep-22 | Feb-22 |
| **Population  description** | Australian parents or caregivers of a child aged 6 months to 3 years. | Fathers or male caregivers with a child aged 6 months to 5 years old who were experiencing socioeconomic disadvantage. | Low-income predominantly Hispanic/Latino families enrolled in a multisite Special Supplemental Nutrition Program for Women, Infants and Children (WIC) in northern Manhattan | Australian fathers experiencing disadvantage | Caregiver-infant dyads with an infant aged 7-9.9 months. Feeding style or food security status were not specified when advertised. |
| **Inclusion criteria** | Participants were eligible to participate if they had a child between 6 months and 3 years of age. They self-identified as experiencing financial hardship by responding yes to the screening question Do you sometimes struggle to pay the bills? | All male caregivers were eligible, sampling was not limited to biological, residential fathers. An affirmative response to the question "Do you sometimes struggle to pay the bills?" was used as an indicator of socioeconomic disadvantage given financial barriers to food security are common. Other eligibility criteria were: (i) at least 18 years of age, (ii) ability to speak and read English, and (iii) the index child did not have any health conditions that affected appetite, feeding or growth. | Mothers (pregnant women and women with infants aged less than 2 years) who could answer questions in English or Spanish. | Participants self-screened using two criteria: (1) being a father or male caregiver of a child aged six months to five years and (2) affirmatively responding to the question Do you sometimes struggle to pay the bills? Additional eligibility criteria included being at least 18 years of age, English proficiency, and the absence of health conditions that affected appetite, feeding, and growth in the index child. | Infant aged 7-9.9 months old.  Caregivers were required to be at least 16 years of age, live in the Auckland or Dunedin regions (main cities in the North and South Islands of New Zealand),  In addition, the infant was not eligible if they had recently taken part in a nutrition intervention study that involved changes to how the infant was fed, and caregivers needed to be able to communicate in English. |
| **Method of  recruitment** | Other: The primary method was through partnerships with child health nurses, community organizations, and charitable groups that support families, where the study was communicated through social media or by displaying hard-copy flyers in areas frequented by families. | Voluntary | Other: Mothers who participated  in the New York City First 1,000 Days Study | Other: 1) paid social media advertisements targeting males interested in parenting, fatherhood, and food;  2) study information posted on relevant Facebook pages;  3) study flyers shared with organizations providing family and child services and food relief services | Recruitment drives via community centres and regular advertising were undertaken in the chosen areas |
| **Total number of  participants** | 29 | 25 | 394 mothers - 113 pregnant women, 281 mothers with infants aged less than 2 years | 264 total participants  - younger child group (<2 years; n = 105) | 604 |
| **Food insecurity definition** | Food insecurity is defined as limited financial, physical, and social access to food of sufficient quality and quantity for a healthy and active life. (Food and Agriculture definition) | Food insecurity refers to reduced physical, social and economic access to sufficient, safe and nutritious food that meets dietary needs and preferences (FAO definition) | Food insecurity, defined by a lack of consistent access to sufficient nutritious food (USDA definition). | Food insecurity occurs when people have inadequate access to food of sufficient quantity and quality to meet dietary needs and preferences (American dietetic association). | Household food insecurity is defined as a ‘limited or uncertain availability of nutritionally adequate and safe foods or limited ability to acquire acceptable foods in a socially acceptable way’ (Anderson, 1990). |
| **Child age Range** | 7 months - 13 years (total child age range) |  |  | 5 months - 23 months | 7-9.9 months |
| **Child age Mean** |  | Age (years), median (IQR) = 2.9 (1.3-4.8) |  | 13.2 months Median (IQR) 9.2-18.3 | 8.4 (SD 0.8) |
| **Child age Other** | At least 1 child within the age range of interest. |  | Median age 6 months | 2 children were slightly outside the age range but had started solids. Both 5 months old (5.3 and 5.4) |  |
| **Child  gender** |  |  | 51% male | 65 (62%) boy  36 (34%) girl | Male 326 (54%) Female 277 (45.9%) |
| **Parent age Range** | 21-43 years |  |  | 29-35 years |  |
| **Parent age Mean** | 32 SD 5.6 years | 35 (SD = 6) |  | 32 years | 32.7 years |
| **Parent age  Other** |  |  | Median age 29 years |  |  |
| **Parent gender** | 28 women and 1 man | male |  | Male | Mostly Female (99% selected relationship to infant as mother) |
| **Household Food Security Status** | Not measured. Participants were eligible if they self-identified as experiencing financial hardship.  Q about the poverty line - below n=10, marginal n=13, above n=6.  Q about subjective financial situation - cannot make ends meet n=1, must cut back n=11, enough, no extras n=14, comfortable with extras n=3.  Appeared as a code created from the data, the phrase itself was not a theme. But characteristics of food insecurity appeared throughout.  Theme - Making trade-offs and sacrifices. Quantity and quality of food was sacrificed to ensure there was enough to share. Mothers sacrificed their intake, reducing quantity, and giving the healthier foods to children.  Buying foods mother and child both like, or prioritising childs foods. Theme - the inescapable impact of covid-19 - "All families referenced the covid-19 pandemic, which magnified and exacerbated the negative effects of hardship." Theme - the unseen mental load - "The possibility of running out of food was a constant concern for many parents." | Not measured. An affirmative  response to the question "Do you sometimes struggle to pay the bills?" was used as an indicator of socioeconomic disadvantage given financial barriers to food security are common was their reasoning.  Appeared as a theme created from the data. Parents talked about: Many fathers protected their child's food access to ensure the child "never go without". Fathers accessed food relief in emergencies.  Socioeconomic disadvantage and food insecurity prompted fathers to adjust feeding strategies to realign mealtime goals with their lived re­ality.  Quality and variety of foods decreased. Focus was on foods that were filling when household budgets were tight.  Theme - Struggling with financial and mental strain, and food insecurity -  Food insecurity was experienced as chronic or transitory... that could be cyclical in nature... These experiences varied in severity, ranging from worrying about food to experiencing hunger and weight loss.  The COVID-19 pandemic further affected local food availability and affordability. Fathers worried about the quality, price, and access. | Household food insecurity was  experienced by 63% of all mothers. Mothers born outside of the US were more likely to experience food insecurity. Measured using the validated 2-item Hunger Vital Sign. | Assessed using 2 measures.  1) A single-item question from the Australian National Health Survey.  2) 18-item USDA HFSSM - adult and child indicators Given as a whole rather than split into child age groups. 37% of households were food insecure according to the Australian NHS single item. With HFSSM 77% of households were food insecure. These are for dichotomized data.  For categorised data:  Household - 10% high food security, 14% marginal, 32% low, 44% very low Adults - 12% high, 17% marginal, 22% low, 49% very low  Children - 52% high and marginal, 46% low, 2% very low | Household food security status was measured using the interviewer-administered ‘food security measurement tool for New Zealand households' (Parnell & Gray, 2014). The tool comprises eight New Zealand food insecurity indicator statements reflecting experiences of household financial constraint over the past 12 months and is a validated measure of household food insecurity in the New Zealand population (Parnell & Gray, 2014).  Households were classified into one of three categories of food insecurity (severely food insecure, moderately food insecure, food secure).  Food secure - 453  Moderately food insecure - 105  Severely food insecure - 46 |
| **Diet** | Parents took strategies to try to protect childs diet such as going to the food bank and borrowing money.  Parents focused on filling meals so their children wouldn't feel hungry  Parents added ingredients to make meals stretch further - rice, pasta, beans.  Parents redefined a good dinner so that basic was not considered bad.  Parents tried to prioritise fruit and veg. | "Fathers felt stress in prioritising necessities, which compromised diet quality and quantity. Processed foods with higher satiety replaced perishable, expensive, healthy foods."  Fathers changed how they accessed foods. Going to the flea market and making foods at home rather than going to the shop.  "Lack of food variety and quality and insufficient quantity for larger households. Some fathers also described the challenge of using unfamiliar foods from hampers."  To control the household budget, fathers stopped offering more expensive, novel, or previously rejected foods and instead prepared more filling and familiar foods. | Of the 183 food insecure infants, 63 (34%) habitually consumed SSB. Infants with food insecurity were 2x more likely to consume SSB compared with infants without food insecurity, even after adjusting for maternal education or foreign-born status.  Infants habitual SSB consumption. Total - 83 (30%). HFI - 63 (34%). No HFI - 20 (20%). P=0.01 |  | At 6 months of age, mothers experiencing severe food insecurity were significantly (p > 0.05) more likely to use commercial baby food pouches frequently for complementary feeding, with almost half (41.3%, n = 19/46) using pouches frequently, compared to much lower proportions for mothers experiencing moderate food insecurity and food secure mothers (26.7%, n = 28/105 and 14.1%, n = 64/453, respectively).  At the current age, almost two-thirds (60.9%, n = 28/46) of mothers experiencing severe food insecurity used pouches frequently. In fact, only 6.5% (n = 3/46) of mothers experiencing severe food insecurity did not use pouches, compared to more than one-quarter (28.3%, n = 128/453) of food secure mothers. The odds of current frequent pouch use in mothers experiencing severe food insecurity were more than five times those of food secure mothers (adjusted OR; 5.70, 95% CI [1.54, 21.01]). Our findings suggest that in the context of food insecurity, the use of commercial baby food pouches may be a convenient means of providing infants with ‘ready-to-eat’ complementary food that is regarded as nutritious and less likely to be wasted.  Mothers experiencing severe food insecurity who currently used pouches frequently also perceived them to be nutritious; specifically because they made it ‘easy to get fruit and vegetables in’ (64%), and ‘easy to get meat in’ (39%).  Three-quarters (75%) of mothers experiencing severe food insecurity used pouches frequently because their infant enjoyed them, suggesting that pouches provided greater certainty in the infant's acceptance of the food. |
| **Feeding practices** | Messy play with food caused tension due to viewing the food as being wasted, and money wasted.  Theme - resilience and being creative  Eating together mattered to some parents. They also saw it as a chance for their child to learn social skills. | There were concerns over food waste amongst fathers, especially with children's fluctuating appetites, food prefer­ences and fussy eating behaviours.. Theme - Paternal feeding practices are driven by values, adversity and emotions.  Many fathers appreciated mealtimes as a setting for family socialisation and promoting child autonomy. Providing a variety of foods and regular meal routines were recognised as important to establish healthy child eating habits. Conversely, some fathers perceived eating as simply the provision of nutrients.  Mealtimes were perceived as an opportunity for reciprocal learning where fathers needed to trust their child rather than enforcing strict rules and linking rewards/punishments to eating. As such, they allowed children to eat to appetite and avoided "forcing kids to eat". Concerns about undereating prompted more monitoring and pressuring behav­iours. Different forms of pressure included setting punishment and using food or non-food rewards  The use of electronic devices varied. Some fathers created no screens rules because of the disruption to eating and socialisation, while others believed eating in front of the TV provided a relaxed atmosphere. Screens were adopted as a tool to get children to behave/eat, keep children at the table or mitigate power struggles in response to fussy eating behaviours despite recognising its futility.  Socioeconomic disadvantage and food insecurity prompted fathers to adjust feeding strategies to realign mealtime goals with their lived re­ality. These practices reduced children's exposure to foods. Despite understanding its implications on child eating behaviours and nutrition, fathers had to place restrictions on what and how much a child could eat, which were non-responsive to their appetite cues.  Fathers reported using less ideal feeding practices due to momentary factors such as parental or child mood. In response to fussy eating or mealtime tantrums, fathers reported employing coercive practices (e.g., squeezing in extra bites) or providing low structure (e.g., child playing and eating on the couch). They relaxed usual rules, catering to the child's food pref­erences or bribing them with food (e.g., dessert if the meal is eaten). These practices were used to avoid fights to reach the end of a meal or relieve their concerns of having "hungry kids". |  | Younger child age group, independent variable food insecurity  Using food to calm (n=81) B = 0.122, p = 0.542. ANOVA = 0.003** Coercive control - Persuasive feeding (n=83) B = 0.552, p = 0.016. ANOVA = 0.005** Coercive control - Parent-led feeding (n=85) B = 0.472, p = 0.030* ANOVA = 0.063 Family meal environment (n=48) B = -0.064 p = 0.819. ANOVA = 0.138  The findings provide some support that food insecurity may elevate the adoption of coercive control practices, especially among the young child group. | Also at 6 months of age, mothers experiencing severe food insecurity were significantly (p < 0.05) less likely to use the full BLW feeding approach than mothers experiencing moderate food insecurity and food secure mothers (2.2%, n = 1/46 vs. 15.2%, n = 16/105 and 13.5%, n = 61/453, respectively), but were more likely to use partial BLW. Thus, the proportions following traditional spoon-feeding were similar in the three groups, ranging from 71.4% to 77.5%.  By the ‘current’ infant age (mean age 8.4 months), the use of full BLW had increased in all groups and there were no longer any statistically significant differences in complementary feeding approaches between the groups. There was no evidence of an association between household food security status and either current partial (e.g., severely food insecure: adjusted OR; 1.11, 95% CI [0.46, 2.66]) or full (e.g., severely food insecure: adjusted OR; 1.03, 95% CI [0.44, 2.43]) BLW in the adjusted models. |
| **Key findings** | Parents of young children aged 6 months to 3 years shared experiences of hardship and resiliency in feeding the family on a limited income in Australia. Despite facing challenges and family tensions, parents demonstrated adaptive strategies and creative food resource management to optimize food availability in the home. Parents experienced a high mental load that needs to be considered when designing programs that support child feeding. | At an intrapersonal level, household food insecurity is a barrier to responsive feeding practices. Living with disadvantage created mental and physical strain and impacted what, when and how fathers fed their children. The results emphasised that paternal feeding involvement and practices are multifactorial, recognising personal, interpersonal, and systemic enablers and barriers. | This study demonstrated that household food insecurity was associated with greater odds of SSB consumption in the first 1,000 days among predominantly Hispanic/Latino WIC-enrolled mothers and young infants. | The novel findings from this research highlight that food insecurity and household chaos were associated with paternal coercive control and autonomy support practices.The findings provide some support that food insecurity may elevate the adoption of coercive control practices, especially among the young child group. | Commercial baby food pouches are a popular method of complementary feeding among food insecure households, with almost two-thirds of mothers who were experiencing food insecurity using pouches frequently.  Mothers in food insecure households who used pouches frequently reported using pouches primarily for reasons of convenience and because they considered them to be nutritious.  Research investigating the impact of frequent pouch use on infant nutrient intake and health is needed to inform advice given to families by health professionals. |
